# T-Pattern Analysis and Cognitive Load Manipulation to Detect Low-Stake Lies: An Exploratory Study

**DOI:** 10.3389/fpsyg.2018.00257

**Published:** 2018-03-02

**Authors:** Barbara Diana, Valentino Zurloni, Massimiliano Elia, Cesare Cavalera, Olivia Realdon, Gudberg K. Jonsson, M. Teresa Anguera

**Affiliations:** ^1^Department of Human Sciences for Education, University of Milano-Bicocca, Milan, Italy; ^2^Department of Psychology, Catholic University of the Sacred Heart, Milan, Italy; ^3^Human Behavior Laboratory, University of Iceland, Reykjavik, Iceland; ^4^Faculty of Psychology, Institute of Neurosciences, University of Barcelona, Barcelona, Spain

**Keywords:** deception detection, cognitive load manipulation, kinesics, analysis of observational data, T-patterns

## Abstract

Deception has evolved to become a fundamental aspect of human interaction. Despite the prolonged efforts in many disciplines, there has been no definite finding of a univocally “deceptive” signal. This work proposes an approach to deception detection combining cognitive load manipulation and T-pattern methodology with the objective of: (a) testing the efficacy of dual task-procedure in enhancing differences between truth tellers and liars in a low-stakes situation; (b) exploring the efficacy of T-pattern methodology in discriminating truthful reports from deceitful ones in a low-stakes situation; (c) setting the experimental design and procedure for following research. We manipulated cognitive load to enhance differences between truth tellers and liars, because of the low-stakes lies involved in our experiment. We conducted an experimental study with a convenience sample of 40 students. We carried out a first analysis on the behaviors’ frequencies coded through the observation software, using SPSS (22). The aim was to describe shape and characteristics of behavior’s distributions and explore differences between groups. Datasets were then analyzed with Theme 6.0 software which detects repeated patterns (T-patterns) of coded events (non-verbal behaviors) that regularly or irregularly occur within a period of observation. A descriptive analysis on T-pattern frequencies was carried out to explore differences between groups. An in-depth analysis on more complex patterns was performed to get qualitative information on the behavior structure expressed by the participants. Results show that the dual-task procedure enhances differences observed between liars and truth tellers with T-pattern methodology; moreover, T-pattern detection reveals a higher variety and complexity of behavior in truth tellers than in liars. These findings support the combination of cognitive load manipulation and T-pattern methodology for deception detection in low-stakes situations, suggesting the testing of directional hypothesis on a larger probabilistic sample of population.

## Introduction

Deception is a matter of everyday life, as several studies have underlined. Turner et al. (1975) estimated that 62% of the statements in everyday general conversations could be somehow classified as deceptive. DePaulo et al. (1996) employed a 1-week diary study to record people’s everyday communication, specifically deceptive communication. Their results suggest that people tell approximately two lies per day on average and that approximately 20 to 33% of our daily interactions are deceptive. Serota et al. (2010) report an average of 1.65 lies in a 24-h period. These data have been supported in two other empirical studies (Hancock et al., 2004; George and Robb, 2008). With a similar methodology, Hancock et al. (2004) observed 26% of our everyday communication to be involving some form of deception, while George and Robb (2008) estimated that 22–25% of our daily communication might be deceptive.

Even though percentages slightly change among different studies, we can affirm deception is a ubiquitous phenomenon which has evolved to become a fundamental aspect of human interaction (O’Sullivan, 2003; Trivers, 2011). Despite prolonged efforts across a broad array of contexts and disciplines, a diagnostic cue to deception has not been found yet (DePaulo et al., 2003; Sporer and Schwandt, 2007; Vrij, 2008).

Two cognitive lie detection approaches emerge from the literature, both relying on the classic Cognitive Capacity Theory (Kahneman, 1973) and adapted from the Cognitive Load Theory (Van Merrienboer and Sweller, 2005). The “mere cognitive load approach” and the “imposing cognitive load approach.”

The first approach (“mere cognitive load”), assumes that the act of lying itself generates observable signs of cognitive load (intrinsic cognitive load). This is also known as the traditional cognitive lie detection approach, based on the work by Zuckerman et al. (1981). Several authors agree in stating that some aspects of lying contribute to the increased mental load (e.g., DePaulo and Kirkendol, 1989; Buller and Burgoon, 1996; DePaulo et al., 2003; Kassin and Norwick, 2004; Kassin, 2005; Vrij et al., 2006; Walczyk et al., 2013).

Lying is not always more cognitively demanding than truth telling (McCornack, 1997). Differences between liars and truth-tellers may be relatively small, and perhaps not readily discernable by observers (Zuckerman et al., 1981; DePaulo et al., 2003), especially when considering low-stake lies. Low-stake lies include pedagogic or white lies, day-to-day polite lies (DePaulo et al., 1980), as well as different kinds of concealment, omission, and evasive messages. On these occasions deceivers and truth-tellers are assumed either to have little to gain (or lose) by being judged deceptive by the addressee or to feel little fear of being caught telling these lies as required by a polite society (Frank and Ekman, 1997). The liar can be at ease in these contexts and does not need particular cognitive demands in generating this kind of deceptive message (Anolli et al., 2002). Conversely, high-stakes lies may have serious effects and consequences for both the deceiver and the deceived. Generally, these kinds of lies are likely to happen in complicated relational situations and in conflicting or face-threatening contexts, such as police interrogations, customs inspections, and high-stake poker games (Frank and Ekman, 1997; Anolli et al., 2002). The speaker has to face up to high cognitive demands since, telling the truth or telling a lie, he has to fabricate a message with the lowest risk of penalty. Many studies focused on the cognitive design of deception in high-stake contexts (Tsiamyrtzis et al., 2007; for an overview see Vrij et al., 2017), while low-stake ones have been scarcely investigated.

The second approach (“imposing cognitive load”), is based on the manipulation of extraneous cognitive load and, for this reason, we think it is particularly suited for low-stakes lies detection. In this perspective, an additional cognitive demand is imposed on individuals to highlight the observable differences between lying and truth telling. Two variations of this method can be identified: in some studies, cognitive demand was increased by making the lie task harder (e.g., Vrij et al., 2008, 2011), while other ones adopted the dual-task paradigm (Baddeley and Hitch, 1974; Baddeley, 1992). For this study, we chose the second option.

Techniques under the heading of dual-task paradigm seek to induce cognitive load selectively on liars not making the lie task harder but rather by altering other aspects of the examination procedure or context. In dual-task paradigm experiments on deception, researchers ask subjects to carry out a secondary task while lying. Because of the additional resources needed for fabricating and telling the lie, people should find dual task more cognitively difficult when they lie than when they tell the truth, and as a result, they should perform worse when they lie. A powerful framework for understanding multi-task interference effect is the Adaptive Executive Control (AEC), which claims that the major sources of interference are in the competition between concurrent tasks for the same perceptual or motor response systems and the executive process performing one task before another (due to its higher priority given the performer’s goals) (Meyer and Kieras, 1997; Meyer et al., 2002). The second task, therefore, will be as more effective as its execution activates the same underlying mechanisms, loading on the same systems used for creating, processing and telling the specific kind of lie being investigated.

As it happens in standard communication, liars are able to arrange a set of different signaling systems to communicate and make their communicative intentions effective, like language, the paralinguistic system, the face and gestures system, gaze, proxemics and the haptic system, or the chronemic system.

Since no diagnostic cue to deception occurs, it could be that a diagnostic pattern does arise when a combination of cues is taken into account (Vrij, 2008). Several studies showed that multimodal data collection could be effective in deception detection. Vrij (2008) claims that, with a combination of four different variables (illustrators, hesitations, latency period, and hand/finger movements) he was able to classify correctly 84.6% of liars and 70.6% of true tellers (Vrij et al., 2000). Jensen et al. (2010), focused on cues extracted from audio, video and textual data, with the aim of building a paradigm for deception detection via a multi-layered model. They reached a classification accuracy of 73.3%, claiming that deception indicators are subtle, dynamic, and transitory, and often elude a human’s conscious awareness. Other studies have shown that between 71 and 78% of correct classifications were made when the researchers investigated a cluster of behaviors (Heilveil and Muehleman, 1981; Vrij et al., 2004; Davis et al., 2005). In other words, more accurate truth/lie classifications can be made if a cluster of non-verbal cues is examined rather than each of these cues are treated separately.

Of course, people can easily control only those patterns that are manifest and have a macroscopic nature, easily readable from the outside time by time. However, patterns in behavior are frequently hidden from the consciousness of those who perform them as well as to unaided observers (Magnusson, 2006). As Eibl-Eibesfeldt (1970) argued, “behavior consists of patterns in time. Investigations of behavior deal with sequences that, in contrast to bodily characteristics, are not always visible.” When the order of events is the only variable considered, the main challenge is to detect the pattern without being distracted by background noise from other events. T-pattern analysis was developed by Magnusson (2000, 2005, 2006) to find temporal and sequential structure in behavior. The term T-pattern stands for temporal pattern; this approach focuses on determining whether arbitrary events sequentially occur within a specified time interval at a rate greater than that expected by chance. In this way, it detects repeated patterns of behavior units coded as events on one-dimensional discrete scales. Temporal pattern analysis and its related software THEME (Magnusson, 2000) have been applied to a great number of research experiments in very different fields. Patterns have been used to describe, interpret and understand phenomena such as deceptive communication (Anolli and Zurloni, 2009; Zurloni et al., 2013, 2016; Diana et al., 2015), animal and human behavior (Kerepesi et al., 2005; Casarrubea et al., 2015), patient–therapist communication in computer assisted environments (Riva et al., 2005), a wide variety of observational and sports studies, such as analysis of soccer team play (Camerino et al., 2012; Cavalera et al., 2015; Diana et al., 2017), motor skill responses in body movement and dance (Castañer et al., 2009) and deception detection in doping cases (Zurloni et al., 2015).

Moreover, patterns of this kind may often be hard or impossible to detect with the well-known statistical methods that are found in major statistical program packages and behavior research software, such as The Observer (Noldus, 1991; Noldus et al., 2000) or GSEQ (Bakeman and Quera, 1995).

Basing on the literature discussed above and results from previous studies (Vrij, 2008; Zurloni et al., 2013, 2016; Burgoon et al., 2014, 2015; Diana et al., 2015), this work proposes an approach to deception detection combining cognitive load manipulation and T-pattern methodology with three objectives: (a) testing the efficacy of dual task-procedure in enhancing differences between truth tellers and liars in a low-stakes situation; (b) exploring the efficacy of T-pattern methodology in discriminating truthful reports from deceitful ones in a low-stakes context; (c) setting the experimental design and procedure for future and follow-up research.

## Method and Data Analysis

### Participants

The convenience sample was initially composed by 46 students, (50% male and 50% female), aged from 21 to 31, born in Italy and living in the same geographic area (inclusion criteria). They were volunteers, contacted through the University’s online study recruiting platform. Due to the exploratory aim of this study, the recruiting lasted 2 months, until 23 males and 23 females fitting the inclusion criteria signed for the experiment. Six students never showed up for the experiment, restricting our final sample to 40.

Instructions on the recruiting platform informed candidates they would participate in an experiment involving their communicative abilities and working memory. At the beginning of the experiment, all participants signed an informed consent to both audio and video recording and authorized the use and processing of personal data; they were also explicitly informed of the possibility to withdraw from the experiment at any time. To increase motivation further, we guaranteed a restitution of results, giving information about their communicative skills.

### Instruments and Materials

– *post hoc* controls and manipulation check instruments, based on cognitive abilities and possible arousal interferences for the participants involved in the experiment (anxiety levels too high or cognitive capacities too distant from the average). For this purpose, we used the State-Trait Anxiety Inventory (STAI; Spielberger, 1983) to measure trait and state anxiety at different times of the experiment. We also used a digit span test and a Corsi test (Mammarella et al., 2008) for short term memory (phonological and spatial).
– a short video clip was made for the experiment, divided in two segments, which were showed separately (see procedure for information about the trials order). The whole video is a no-dialog story about four characters organizing a bank robbery and committing it. The video is silent because we wanted to avoid having participants use words from the characters and to let them free in the interpretation and accounting of the story (no violent acts or emotionally involving events are depicted, in order to avoid arousal interference). The video was cut approximately in half, so that each segment’s length would be about the same as the other and contain the same number of events.
– dual task procedure. In the experimental condition, participants were asked, on two occasions, to memorize a series of four sentences. The two series are different, balanced to contain the same number of words and the same syntactic structure. Participants were told to remember these sentences since they could be asked to recall them at any time during the experiment. This extra memory task was added to produce interference in visual-spatial memory.
– observation instrument: the behavior categories we identified refer to self-contact gestures (ADAPTOR in the coding grid), illustrator gestures (ILLUSTRATOR), rhythmic gestures (RHYTHMIC), emblems or symbols (SYMBOL), leg movements (LEG), feet movements (ONLY_FOOT), finger movements (ONLY_FINGERS). These behaviors have been chosen according to what emerged in our previous study (Zurloni et al., 2013, 2016), in other studies found in literature (Caso et al., 2006), and also according to what was affirmed by Anolli (2012) concerning the scarcity of distal movements compared to proximal ones during deception, as well as the results obtained by Burgoon et al. (2014) on kinesic T-patterns related to deception. The objective was to create a coding grid that would satisfy the need to simplify interpretation of patterns while identifying specific components in patterns that could be more relevant, when combined, than the configurations tested until now.
– Research Randomizer -Version 4.0- computer software (Urbaniak and Plous, 2013), to randomize the order of trials;
– Behavior Coder software, for manually coding non-verbal behaviors from video sources;
– SPSS 22 software, for statistical analysis of behavior frequencies and pattern data.
– Theme 6.0^1^ software for T-pattern detection. This software detects the temporal structure of data sets, revealing repeated patterns (T-patterns) that regularly or irregularly occur within a period of observation. It allows the detection of repeated temporal patterns even when multiple unrelated events occur in between components of the patterns.

### Procedure

This study was carried out at the University of Milano-Bicocca in an audio-isolated laboratory room equipped with four cameras, set to video-record participants’ full-lengths and close-ups. The cameras were connected to a 2-channel quad device (split-screen technique).

The participants, males and females, were assigned to conditions using a procedure (control condition for the first male participant, experimental for the second and so on; the same with the female group) designed to have the same number of participants, balanced for gender, per group (as near as possible).

The whole experiment, in both conditions, lasted 40–50 min. After signing the informed consent form, all participants filled in the STAI_T inventory. After this, the experimenter administered them a digit span memory test and a Corsi test for spatial memory.

The experiment, in general, consisted of watching two segments of a video (see Instruments and Materials) and then report to a confederate the truth about the content of one segment and lie about the content of the other. The order of trials has been randomized within the two conditions with a randomization software (Research Randomizer, Urbaniak and Plous, 2013), so that half of the participants would lie about the first video, while the other half about the second one (see **Table 1**).

**Table 1:** Number of participants per trial within conditions.

	Control	Experimental
Trial 1^∗^	9	10
Trial 2^∗∗^	9	9

*^∗^Trial 1: Truthful report for the first video and deceptive for the second. ^∗∗^Trial 2: Deceptive report for the first video and truthful for the second.*

In the control condition, before the first video, participants are asked to watch and pay attention to every detail, explaining that the next part of the experiment will concern that particular video. After watching the video, the examiner asks them to fill out a STAI_S inventory, to measure state anxiety (and verify possible changes in arousal provoked by watching the video or other conditions). Then, depending on the trial, they are asked to report what they saw in the video or to lie about what they saw in the video to an interlocutor who, to their knowledge, does not have prior information about its content. Participants are given a list of things they can use in their report (if they have to lie, there are examples of details that can be changed, such as the number or gender of the characters, their features, their actions, etc.). They are given 5 min alone to recall and organize their report (mentally); after this, the confederate enters the room and is introduced to the participant as a fellow participant. The examiner exits the room and starts the recording. An audio signal cues the participant to start telling his/her report to the interlocutor, who does not participate in the conversation. After this part, the examiner ends the recording, goes back to the room and restarts the same routine with the second video (second part of the story). Participants are given the same instructions as before, adding the information that the interlocutor does not know which part of their story is made up but knows that one part is. This was necessary so they would not have to justify inconsistencies with their first report, since they are expected.

In the experimental condition (cognitive load manipulated), the procedure is the same but participants have to perform a dual task; before starting each of their reports, they are given a list of 4 numbered sentences to memorize and are told that they will have to recall them (when asked by the examiner) at random times during their report. They do not know when they will be interrupted or which sentence will be asked to recall, nor how many times this will happen. To enhance interference, instructions suggest to keep recalling the sentences mentally during the whole report. After the instructions, participants are given two extra minutes to memorize the sentences. Then, like in the control condition, the interlocutor enters the room and, after an audio signal, participants start their report.

At the end of the two segments, the examiner tells participants that the experiment is over and answers any question they might have about the procedure or the study.

### Data Analysis

The memory span and anxiety tests we used for exclusion criteria did not show outliers. Nonetheless, 3 participants out of the initial 40 had to be excluded for technical problems with the recordings, restricting our sample to 37 participants and 74 reports (data from the 37 participants included in the analysis were available for all the measures considered, see **Table 1**). The videos were coded on Behavior Coder software by two coders, using a blind coding procedure. The occurrences of each event-type within the selected observation period form the so called T-dataset’.

To assess inter-rater reliability of the T-dataset, Cohen’s Kappa was calculated on 10% of the encodings. Although differing through categories, inter-coder reliability was found to be good to satisfactory (ranging from 0.78 to 0.90; *p* < 0.05). When disagreements were identified or the agreement was not perfect, the specific cases were discussed and agreed on by both coders.

#### Single Cues

We carried out a descriptive analysis of the behaviors’ frequencies coded through Behavior Coder, using SPSS (22). The aim was to show shape and characteristics of the distributions. Next, we carried out Mann-Whitney and Wilcoxon Signed Rank Tests, as a guideline for interpreting data and exploring differences between groups.

#### T-Pattern Detection

T-datasets were then analyzed with Theme 6.0^2^ for T-pattern detection. A T-pattern is essentially a combination of events where the events occur in the same order, with the consecutive time distances between consecutive pattern components remaining relatively invariant, regardless of the occurrence of any unrelated event in between them (Magnusson, 2005).

The 74 datasets were analyzed with THEME software to search for patterns and describe behavior structure and complexity (number of unique T-patterns, mean T-patterns’ lengths and levels) in the truth and deception data, exploring differences between groups in the control and experimental conditions. The software allows to set statistical parameters for the pattern detection, according to research aims and scope. For this study, the threshold pattern significance was set to *p* < 0.005 and the minimum number of pattern occurrences was set to 2 (chosen based on mean length of the observation period, 2 min; Zurloni et al., 2015).

A descriptive analysis on T-pattern frequencies was carried out to show shape and characteristics of the distributions; Mann–Whitney and Wilcoxon Signed Rank Tests as a guideline for interpreting data and exploring differences between groups.

An in-depth analysis on more complex patterns was performed to get qualitative information on the behavior structure expressed by the participants. We chose to consider the more complex patterns because they represent the highest level of organization expressed by the participants in the two conditions.

## Results

### Single Cues

**FIGURE 1:**
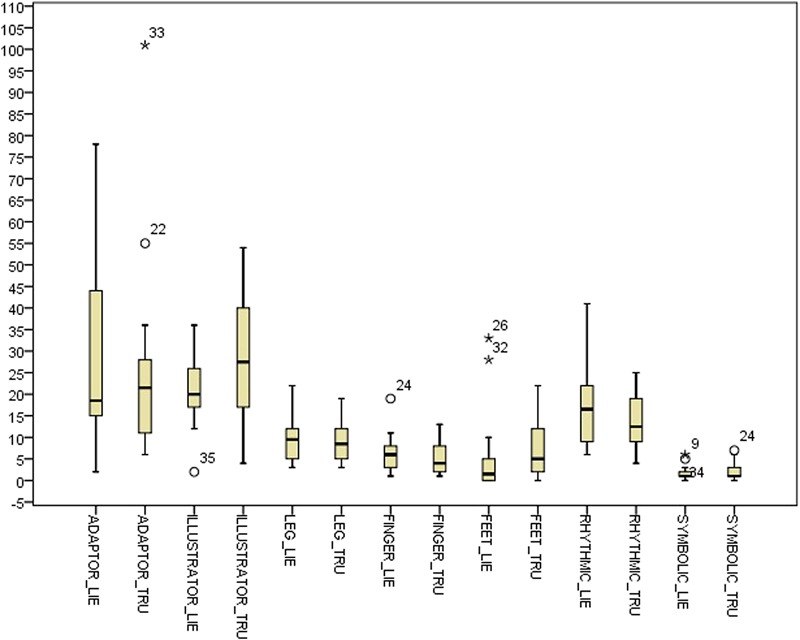
Box-plot for single cues in the control condition.

**FIGURE 2:**
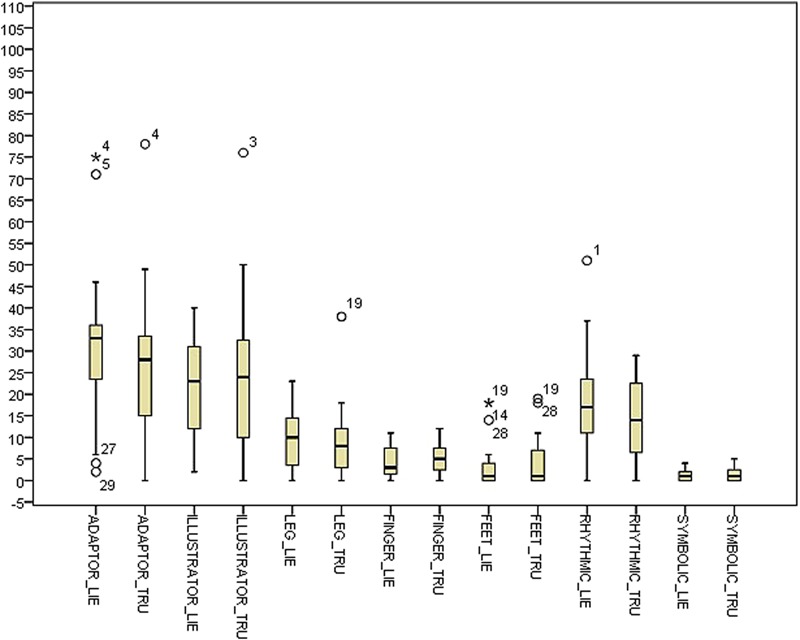
Box-plot for single cues in the experimental condition.

**Table 2** shows descriptive statistics for the difference between the deceptive and truthful reports for the single cues within the control condition. **Table 3** shows the corresponding statistics within the experimental condition. **Table 4** and **Figure 1** show descriptive statistics of single cues in truthful and deceptive reports within the control condition, while **Table 5** and **Figure 2** show descriptive statistics of single cues in truthful and deceptive reports within the experimental condition.

**Table 2:** Descriptive statistics for the difference between the deceptive and truthful reports for the single cues within the control condition.

Control condition	Mean	Mean standard error	Median	Standard deviation	Minimum	Maximum	Int.Q range	First quartile	Third quartile	Skewness	Kurtosis
ADAPTOR_LT	1.67	6.34	-1.50	26.91	-79.00	51.00	130.00	-8.50	22.25	-1.21	4.38
ILLUSTRATOR_LT	-6.78	3.54	-7.00	15.02	-26.00	32.00	58.00	-16.75	0.25	0.97	1.43
LEG_LT	0.17	1.22	-0.50	5.16	-9.00	10.00	19.00	-4.00	3.75	0.21	-0.56
FINGER_LT	1.44	0.94	0.50	3.99	-5.00	10.00	15.00	-1.25	5.00	0.36	-0.29
FEET_LT	-1.33	1.92	-1.50	8.15	-21.00	21.00	42.00	-4.25	0.25	0.51	4.26
RHYTHMIC_LT	3.33	1.69	2.50	7.19	-7.00	18.00	25.00	-2.00	7.25	0.65	-0.22
SYMBOLIC_LT	-0.39	0.51	0.00	2.15	-5.00	4.00	9.00	-1.25	1.00	-0.39	0.71

**Table 3:** Descriptive statistics for the difference between the deceptive and truthful reports for the single cues within the experimental condition.

Experimental condition	Mean	Mean standard error	Median	Standard deviation	Minimum	Maximum	Int.Q range	First quartile	Third quartile	Skewness	Kurtosis
ADAPTOR_LT	4.10	3.22	4.00	14.03	-35.00	24.00	59.00	-5.00	16.00	-0.99	2.03
ILLUSTRATOR_LT	-3.47	3.33	2.00	14.53	-36.00	16.00	52.00	-17.00	7.00	-0.88	-0.14
LEG_LT	0.63	2.02	0.00	8.83	-24.00	15.00	39.00	-1.00	5.00	-1.01	2.67
FINGER_LT	-0.79	1.00	0.00	4.37	-8.00	7.00	15.00	-5.00	2.00	0.09	-0.48
FEET_LT	-0.26	0.75	0.00	3.28	-8.00	7.00	15.00	0.00	1.00	-0.57	1.86
RHYTHMIC_LT	4.37	1.92	3.00	8.35	-8.00	23.00	31.00	-4.00	11.00	0.26	-0.38
SYMBOLIC_LT	-0.21	0.36	0.00	1.58	-4.00	2.00	6.00	-1.00	1.00	-1.39	1.98

**Table 4:** Descriptive statistics for the single cues in truthful and deceptive reports within the control condition.

Control condition	Mean	Mean standard error	Median	Standard deviation	Minimum	Maximum	Int.Q range	First quartile	Third Quartile	Skewness	Kurtosis
ADAPTOR_LIE	27.61	4.75	18.50	20.17	2.00	78.00	29.75	14.50	44.25	1.05	0.65
ADAPTOR_TRUTH	25.94	5.25	21.50	22.26	6.00	101.00	18.50	10.50	29.00	2.51	7.64
ILLUSTRATOR_LIE	20.94	1.95	20.00	8.29	2.00	36.00	10.00	16.50	26.50	-0.07	0.81
ILLUSTRATOR_TRUTH	27.72	3.22	27.50	13.66	4.00	54.00	24.25	16.00	40.25	-0.02	-0.54
LEG_LIE	9.39	1.14	9.50	4.85	3.00	22.00	7.50	4.75	12.25	0.80	1.14
LEG_TRUTH	9.22	1.11	8.50	4.72	3.00	19.00	7.50	5.00	12.50	0.74	-0.30
FINGER_LIE	6.44	1.01	6.00	4.29	1.00	19.00	5.25	3.00	8.25	1.30	3.41
FINGER_TRUTH	5.00	0.85	4.00	3.60	1.00	13.00	6.25	2.00	8.25	0.88	-0.13
FEET_LIE	5.39	2.25	1.50	9.55	0.00	33.00	5.00	0.00	5.00	2.39	4.95
FEET_TRUTH	6.72	1.53	5.00	6.48	0.00	22.00	10.25	1.75	12.00	1.12	0.60
RHYTHMIC_LIE	17.22	2.17	16.50	9.19	6.00	41.00	13.75	8.75	22.50	0.95	1.05
RHYTHMIC_TRUTH	13.89	1.62	12.50	6.88	4.00	25.00	11.50	8.50	20.00	0.31	-1.00
SYMBOLIC_LIE	1.67	0.39	1.00	1.64	0.00	6.00	1.25	0.75	2.00	1.50	2.24
SYMBOLIC_TRUTH	2.05	0.49	1.00	2.10	0.00	7.00	2.25	0.75	3.00	1.20	0.68

**Table 5:** Descriptive statistics for the single cues in truthful and deceptive reports within the experimental condition.

Experimental condition	Mean	Mean standard error	Median	Standard deviation	Minimum	Maximum	Int.Q range	First quartile	Third quartile	Skewness	Kurtosis
ADAPTOR_LIE	31.16	4.49	33.00	19.56	2.00	75.00	15.00	22.00	37.00	0.62	0.85
ADAPTOR_TRUTH	27.05	4.25	28.00	18.54	0.00	78.00	21.00	13.00	34.00	0.95	1.92
ILLUSTRATOR_LIE	21.50	2.81	23.00	12.25	2.00	40.00	21.00	11.00	32.00	-0.10	-1.29
ILLUSTRATOR_TRUTH	24.95	4.17	24.00	18.20	0.00	76.00	23.00	10.00	33.00	1.10	2.26
LEG_LIE	9.84	1.83	10.00	7.99	0.00	23.00	12.00	3.00	15.00	0.49	-1.11
LEG_TRUTH	9.21	2.00	8.00	8.73	0.00	38.00	9.00	3.00	12.00	2.09	6.02
FINGER_LIE	4.16	0.79	3.00	3.47	0.00	11.00	7.00	1.00	8.00	0.57	-0.93
FINGER_TRUTH	4.95	0.78	5.00	3.41	0.00	12.00	6.00	2.00	8.00	0.24	-0.69
FEET_LIE	3.89	1.37	1.00	6.00	0.00	18.00	5.00	0.00	5.00	1.78	1.94
FEET_TRUTH	4.16	1.42	1.00	6.18	0.00	19.00	8.00	0.00	8.00	1.53	1.28
RHYTHMIC_LIE	18.95	2.89	17.00	12.59	0.00	51.00	14.00	10.00	24.00	0.92	1.01
RHYTHMIC_TRUTH	14.58	2.22	14.00	9.68	0.00	29.00	17.00	6.00	23.00	-0.05	-1.45
SYMBOLIC_LIE	1.16	0.29	1.00	1.26	0.00	4.00	2.00	0.00	2.00	1.16	0.91
SYMBOLIC_TRUTH	1.37	0.36	1.00	1.57	0.00	5.00	3 .00	0.00	3.00	0.94	-0.11

Results highlight some differences between conditions, especially regarding the distribution of ADAPTOR gestures. In deception data, the median increases from 18.50 in the control condition to 33.00 in the experimental one, while the data dispersion decreases (inter-quartile range: 29.75 in the control condition to 15.00 in the experimental one). Considering data distribution within the control condition, ILLUSTRATOR gestures were more frequently observed in truthful reports (median 27.50) rather than deceptive ones (median 20.00). In the experimental condition, RHYTHMIC gestures were more frequent in deceptive reports (median 17.00), rather than in truthful ones (median 14.00). Independent samples Mann–Whitney *U* Test seems to show no differences in distributions across the two conditions for all the considered indexes. Differences between truth tellers and liars were explored within groups using related samples Wilcoxon Signed Rank Test (Wilcoxon, 1945). Results in the control condition seem to show no differences except for the ILLUSTRATOR behavior (*p* = 0.047), which appeared to be less present in deception than in truthful reports (see **Table 5**).

Results in the experimental condition show a difference in the distributions of RHYTHMIC gestures (*p* = 0.033), more present in deceptive reports than in sincere ones (see **Table 5**).

### T-Pattern Analysis

Descriptive statistics and distributions of T-patterns in the control condition are presented in **Table 6**, while the experimental condition data are presented in **Table 7**.

**Table 6:** Descriptive statistics for T-pattern data in the control condition.

Control condition	Mean	Mean standard error	Median	Standard deviation	Minimum	Maximum	Int.Q range	First quartile	Third quartile	Skewness	Kurtosis
LENGTH_LT	5.65	0.25	5.51	1.06	4.50	8.71	1.08	4.82	5.90	1.63	3.18
LEVEL_LT	3.27	0.17	3.15	0.73	2.50	3.15	0.91	2.71	3–61	1.34	1.65
UNIQUEPAT_LT	19.83	2.62	18.00	11.12	4.00	45.00	13.50	12.00	25.50	0.96	0.34

**Table 7:** Descriptive statistics for T-pattern data in the experimental condition.

Experimental condition	Mean	Mean standard error	Median	Standard deviation	Minimum	Maximum	Int.Q range	First quartile	Third quartile	Skewness	Kurtosis
LENGTH_LT	5.33	0.32	5.19	1.41	2.00	7.97	1.94	4.33	6.27	-0.22	0.66
LEVEL_LT	3.03	0.21	3.14	0.91	1.00	4.40	1.44	2.33	3.78	-0.36	-0.27
UNIQUEPAT_LT	17.84	4.96	14.00	21.62	1.00	100.00	13.00	6.00	19.00	3.33	12.72

**FIGURE 3:**
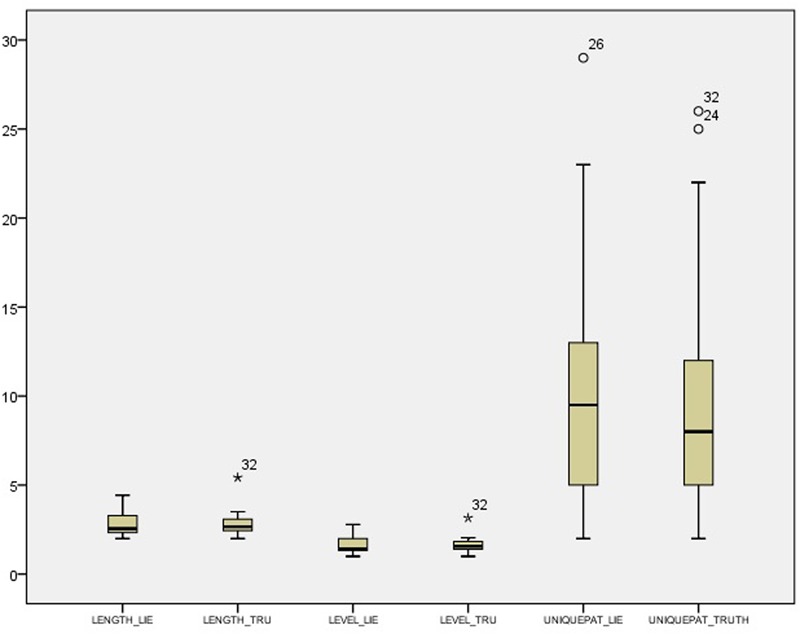
Box-plot for T-pattern data in the control condition.

**FIGURE 4:**
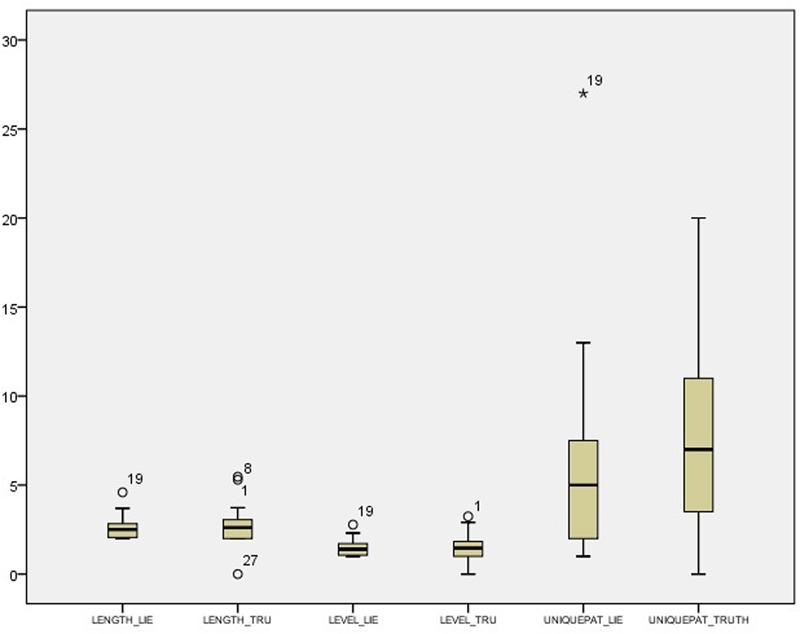
Box-plot for T-pattern data in the experimental condition.

Descriptive statistics and distributions of T-patterns of truthful and deceptive reports in the control condition are presented in **Table 8** and **Figure 3**, while the experimental condition data are presented in **Table 9** and **Figure 4**. Independent samples Mann–Whitney *U* Test suggest a difference in the distributions of unique patterns between the two conditions (*p* = 0.026). In fact, while the control condition shows no differences between truthful and deceptive data (in number of unique T-patterns, mean T-patterns’ lengths and levels), the experimental condition shows a difference in terms of unique patterns between truth tellers and liars (Wilcoxon’s test, *p* = 0.036). In particular, the number of unique patterns is substantially higher in truthful reports than in deceptive ones with a less dispersion in data distribution (see **Table 9**).

**Table 8:** Descriptive statistics for T-pattern data in truthful and deceptive reports within the control condition.

Control condition	Mean	Mean standard error	Median	Standard deviation	Minimum	Maximum	Int.Q range	First quartile	Third quartile	Skewness	Kurtosis
LENGTH_LIE	2.79	0.16	2.55	0.70	2.00	4.43	1.07	2.31	3.39	0.99	0.06
LENGTH_TRUTH	2.86	0.18	2.66	0.76	2.00	5.42	0.67	2.43	3.10	2.41	7.54
LEVEL_LIE	1.62	0.12	1.40	0.50	1.00	2.78	0.71	1.31	2..02	0.93	0.13
LEVEL_TRUTH	1.65	0.11	1.56	0.47	1.00	3.15	0.47	1.38	1.86	1.87	5.49
UNIQUEPAT_LIE	9.83	1.65	9.50	7.01	2.00	29.00	8.25	4.75	13.00	1.51	2.48
UNIQUEPAT_TRUTH	10.00	1.71	8.00	7.24	2.00	26.00	7.25	5.00	12.25	1.35	0.85

**Table 9:** Descriptive statistics for T-pattern data in truthful and deceptive reports within the experimental condition.

Experimental condition	Mean	Mean standard error	Median	Standard deviation	Minimum	Maximum	Int.Q range	First quartile	Third quartile	Skewness	Kurtosis
LENGTH_LIE	2.62	0.16	2.50	0.69	2.00	4.59	1.00	2.00	3.00	1.56	2.59
LENGTH_TRUTH	2.71	0.28	2.61	1.22	0.00	5.47	1.27	2.00	3.27	0.61	2.04
LEVEL_LIE	1.50	0.11	1.40	0.49	1.00	2.78	0.86	1.00	1.86	1.12	1.01
LEVEL_TRUTH	1.53	0.17	1.46	0.74	0.00	3.24	1.00	1.00	2.00	0.57	1.13
UNIQUEPAT_LIE	6.05	1.39	5.00	6.07	1.00	27.00	6.00	2.00	8.00	2.49	7.76
UNIQUEPAT_TRUTH	11.79	4.61	7.00	20.10	0.00	92.00	8.00	3.00	11.00	3.90	16.15

**FIGURE 5:**
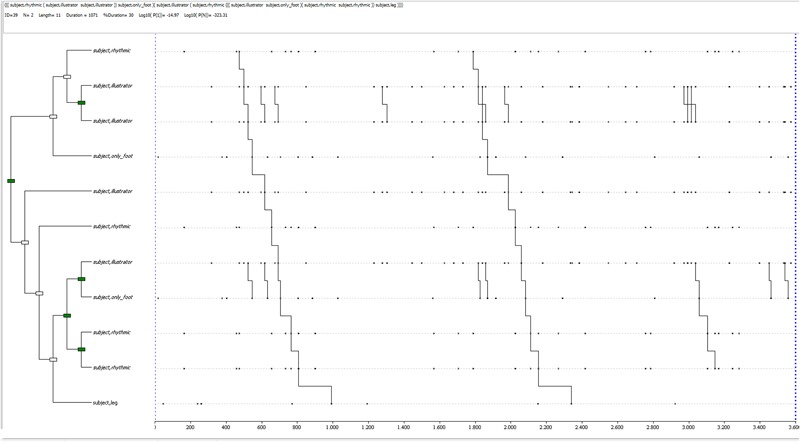
T-pattern extracted from a truthful dataset in the control condition. The events occurring begin with a rhythmic gesture, linked to a sub-pattern made of two illustrator gestures, followed by feet movement; then, another illustrator gesture is followed by a rhythmic one, a sub-pattern including an illustrator gesture and feet movement, another sub-pattern made of rhythmic gestures, to finally end with legs movement. This pattern has a length of 11 event-types, 6 levels and it occurs twice during the whole observation period.

The most distinctive patterns for both conditions have also been qualitatively analyzed. In the control condition, there are few quantifiable differences in detected T-patterns, but it is possible to notice a trend for half of the participants, showing less complex patterns in deceitful accounts and more complex ones in truthful accounts. An example is shown in **Figure 5**^3^, relative to a sincere report in control conditions: it is a complex pattern, characterized by 6 levels, 11 event-types for its length, compared to an average in control conditions of 1.6 levels (*SD* = 0.5) and 2.86 (*SD* = 0.76) event-types. It is composed of rhythmic, illustrator gestures, feet and leg movements. It occurs twice during the whole observation period. Some “blocks” (or sub-patterns) included in this T-pattern are also identified by the software (singularly) and appear next to the complete pattern, occurring often and involving illustrator gestures, mostly.

**FIGURE 6:**
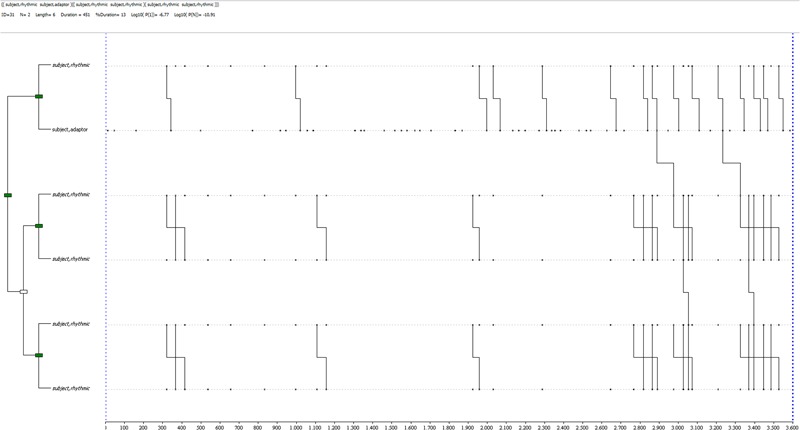
T-pattern extracted from a deception dataset in the control condition. The events occurring begin with a rhythmic gesture, linked to a self-contact gesture, then followed by 2 sub-patterns of linked rhythmic gestures. This pattern has a length of 6 event-types, 3 levels and it occurs twice during the observation period.

A deception-related T-pattern for the control condition by the same participant is shown in **Figure 6**: it is a complex pattern (3 levels, 6 events, with a mean in control deceptive reports of 1.6 levels and 2.8 events), characterized by an alternation of rhythmic and self-contact gestures. It occurs twice during the observation period, toward the end, although different combinations of the same behaviors occur in earlier sections of the observed period.

In experimental conditions, the qualitative evaluation of patterns confirms the lack of richness suggested by the exploratory analysis in deception: T-patterns are generally simple; in some cases, the most complex one is only made by 2 different events. In more complex cases, repetition of gestures of the same category are found, linked in sub-patterns of this kind. Rhythmic gestures are identified in many deception-related patterns.

**FIGURE 7:**
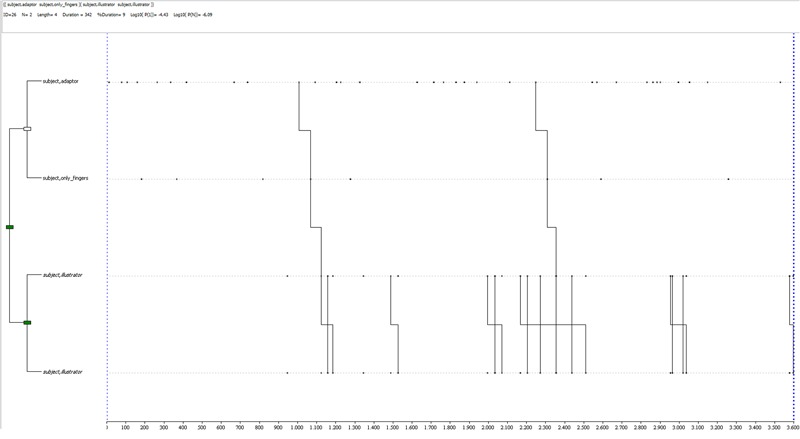
T-pattern extracted from a truthful dataset in the experimental condition. The events occurring begin with a self-contact gesture linked to finger movement (not a gesture), and two illustrator gestures. This pattern has a length of 4 event-types, 2 levels and it occurs twice during the observation period.

The truth condition shows variety in T-pattern compositions, with a general trend toward a complex and varied non-verbal behavior, similar to the control condition patterns. An example is shown in **Figure 7**: a complex pattern, 2 levels and 4 event-types, with a general mean in its group of 1.53 levels (*SD* = 0.74) and 2.71 event-types (*SD* = 1.21). It is made of two sub-patterns including self-contact gestures, finger movements and illustrator gestures.

**FIGURE 8:**
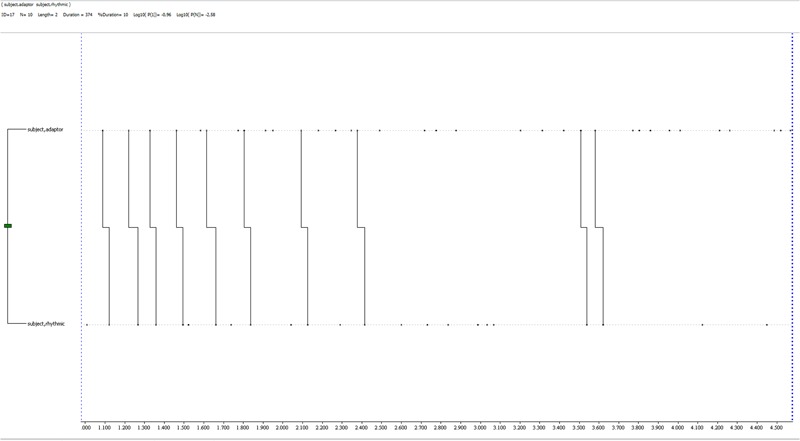
T-pattern extracted from a deception dataset in the experimental condition. It is made of two event-types only, a self-contact gesture and a rhythmic one, both very close to each other. It has a 2 event-types length, 1 level and it occurs 10 times during the observation period.

A T-pattern related to deception reports by the same participant is shown in **Figure 8**: it is a very simple one (1 level, 2 event-types, with a general mean of 1.5 levels and 2.6 events), being made of an alternation of self-contact and rhythmic gestures. It occurs 10 times during the observation period.

## Discussion and Conclusion

Research on deception detection has been focused for a long time on the identification of single unmasking cues, while there have been few studies where deceptive behavior has been observed in a temporal and sequential structure perspective (Vrij, 2008; Burgoon et al., 2015). T-pattern analysis allowed to identify repeated patterns of behavior with different qualities and quantities between deception and truth.

The frequencies observed for single cues suggest that cognitive load manipulation did not affect the occurrence of specific behaviors, except for the adaptor gestures, which clearly decrease in the experimental condition, especially when lying. The decrease in data dispersion could be an effect of cognitive load manipulation. Adaptors (self-contacts and self-manipulations) are self-regulating gestures, that can increase with the increase in emotional or cognitive load (e.g., Vrij et al., 2008). Exploring differences within conditions, illustrator gestures seem to occur more in the truth excerpts of the control group (the less cognitively demanding setting). The decrease of illustrator gestures occurrences during deception has been discussed and linked to the intrinsic cognitive load increase by DePaulo et al. (2003), and by Sporer and Schwandt (2007) in their meta-analyses; our findings seem to confirm that direction. In the experimental condition, rhythmic gestures were found to be more present in deceptive reports than in truthful ones. It is interesting to underline how this supports what was observed by Caso et al. (2006) in a study which used an experimental variable such as the rise of suspicion level, operationalized in a phase of the interview where the interlocutor directly accused the participant to be lying. The cognitive load manipulation condition used in our study could have produced the same result of the invasive interview used in the study by Caso et al. (2006). In conclusion, the behaviors identified in this analysis have already been found to be intrinsic of a cognitively demanding condition; in fact, these could be the effects of the increased arousal related to cognitive overload.

T-pattern frequencies show that the distribution of unique patterns seems to be affected by cognitive load manipulation. These data have to be confirmed by further studies (since the *p-value* was here used as a guideline), but it is interesting to notice that the dual-task procedure interfered on the behavior structure and variety rather than on single behavior’s occurrences. As for the control condition, our data did not show any relevant difference between truth and deception in terms of unique patterns, mean lengths and levels. This result, although to be confirmed, is in accordance with what emerged from literature and supports using techniques that interfere with cognitive load; in fact, the cognitive effort demanded by “low stakes” lying could have been not enough to produce observable effects on non-verbal behavior. In the experimental condition, instead, a difference seems to exist between truth and deception in terms of unique T-patterns, with a higher number of them in truthful reports than in deceptive ones. No differences were found in the number of levels and lengths of T-patterns, although we think that this result might be affected by the length of the observation period. In general, it appears that the cognitive effort related to dual task affected non-verbal behavior variability and richness, making it more stereotypical and “mechanical” (Zurloni et al., 2015).

Qualitative in-depth analysis of detected T-patterns has shown a wide range of behaviors exhibited in patterns of all conditions. It is clear that, in control conditions, differences in non-verbal behavior between lying and truth-telling are subtle or very hard to interpret. For some participants (less than half of the sample), deception and truth-telling were characterized by similar patterns, while for others there is a decrease in complexity while lying (described by the lower mean of lengths, levels, and the number of unique patterns). These differences are not systematic enough to be ascribed to a general rule, although we do not expect them to be, since the huge amount of studies carried out in recent years produced similar results (DePaulo et al., 2003). We can speculate that the lying task might have elicited different responses because of different factors, among them a stronger or weaker inclination to deception (Anolli, 2012), or a more specific advantage or disadvantage caused by the cognitive capabilities used to lie (such as working memory, Baddeley, 1992). As suggested by findings above, in the control condition, many truth-related patterns are characterized by one or more illustrator gestures. In literature, illustrator gestures have been linked to sincerity and rhythmic gestures to deception (DePaulo et al., 2003; Caso et al., 2006). Finding these data in the structures detected by THEME contributes to support its usefulness in observational studies on deception. In the experimental condition, patterns detected by the software clearly indicate the difference between truth telling and lying conditions. In the latter one, in fact, structures of minimal complexity are detected, often as a chain of occurrences of the same kind of gesture. In many cases, patterns related to deception include rhythmic gestures. Caso et al. (2006), in their study on sincerity and deception based on the gestures’ frequencies, have observed the same increase in rhythmic gestures, and we believe a cognitive load increase could explain both results. Patterns related to truth-telling in the experimental condition show a certain variety in composition, similar in its abnormal distribution of frequencies to patterns related to the control condition. These patterns are still less complex than the ones detected in the control condition, but for some participants they are similar to the first ones, including numerous illustrator gestures.

This exploratory study gave results which are in line with findings from our previous studies (Zurloni et al., 2013, 2015), and with the picture described by analyzing existent literature (DePaulo et al., 2003; Sporer and Schwandt, 2007; Vrij, 2008; Burgoon et al., 2014, 2015). Overall, THEME proves to be an effective tool for discriminating truth-telling and deceptive reports in manipulated cognitive load conditions, and the dual-task procedure seems to be effective in this sense. Differences that are not detectable in terms of single cues emerged within the structure of behavior, which, as we have discussed, seems to result less rich and more stereotypical in deception.

### Limits and Future Directions

This work had its main limitations in size and characteristics of the sample but it allows us to proceed in further research, aiming at testing directional hypotheses and confirming these findings. A stratified sampling and a longer observation period will be crucial to enhance the validity of all data extracted by THEME software. The procedure used to assign participants to conditions was meant to make the groups as equivalent as possible, despite the small sample size, but may have provoked biases according to personal characteristics of participants, for instance their motivation level (generally, the participants who enroll first to an experiment are also the most motivated). Manual coding could be an obstacle due to time-consuming practices and may have had negative effects on data quality. New technologies can help with this issue, with automatic data quality control integrated within the observation software (e.g., Castañer et al., 2017) or automatic extraction of relevant data from the source [even motor information, such as facial expressions, gestures and movement, etc. (Zhang, 2012)]. Common examples are motion capture devices like Kinect (e.g., Yu et al., 2011) wearable sensors for the extraction of biofeedback data or unobtrusive techniques like thermal imaging (Pavlidis et al., 2002; Dcosta et al., 2015). Machine learning algorithms, for example (Bartlett et al., 2005) can extract information from video or audio sources and process them through advanced algorithms that can automatically code facial expressions, body movement, typical gestures, emotions, glance direction and tone of speech. All this cues, if collected in a systematic manner, give access to a large-scale analysis, both from an observational and a statistical point of view. THEME software can work with all kind of data or events detected in a particular moment in time, making the potential applications to include a large range of sources. The experimental procedure could be improved, for example adding a naïve interlocutor and considering the interaction as moderator of behaviors. The observation instrument built for this study could be enriched with behavior cues from other systems, such as head movements or facial expressions; although, it is fundamental to keep a balance between the exhaustivity of observed behaviors and the interpretability of results.

### Impact

Deception is a ubiquitous phenomenon, regulated by the same processes used in “standard” communication. Furthermore, lying implicates an additional use of cognitive resources which, in “low stakes” conditions (low risk, low gain), common to daily life and to which humans are more “practiced,” is an insignificant (or at least, not currently measurable) amount. We believe that, with cognitive approaches, a step forward was made in studying deception as a standard communication phenomenon: manipulating cognitive load allows unveiling differences otherwise inaccessible to analysis, because they are usually intrinsic to communication processes. In our experiment, THEME software was able to detect differences in behavior structure when the cognitive load experienced from participants was the highest. Although these findings need to be confirmed (a physiologic and a self-report measure of the cognitive effort could be important), it would be interesting to explore if the intrinsic higher cognitive load characterizing high-stakes deception could be enough to allow a detection without manipulation. If proved, the transferability and application of this methodology in real life contexts could be easier and potentially include several research and interventions areas, such as public security monitoring (frontiers, airports, stations, etc., Burgoon et al., 2014) or the detection of illegal and/or dangerous behaviors, for instance doping in sport (Zurloni et al., 2015).

## Ethics Statement

At the time we collected data for this study, the IRB approval, in our institution, was not mandatory for minimum-risk studies. For this reason, the present study was conducted according with the ethical principles stated by the Association of Italian Psychologists and the general principles stated in The Federal Policy for the Protection of Human Subjects (or the “Common Rule”), defining “minimum” the risk in which “the probability and magnitude of harm or discomfort anticipated in the research are not greater in and of themselves than those ordinarily encountered in daily life or during the performance of routine physical or psychological examinations or tests” (45 CFR 46.102). The literature agrees in considering low-stakes deception to be a daily-life phenomenon.

## Author Contributions

BD and VZ contributed in method development, study designing, data analysis, and paper writing. ME contributed in data acquisition and coding, data analysis, and paper writing. CC contributed in study designing and data acquisition and coding. OR contributed in study designing and data analysis. GJ contributed in method development and data analysis. MTA contributed in method development and paper writing. All authors made suggestions and critical reviews to the initial draft and contributed to its improvement until reaching the final manuscript, which was read and approved by all authors.

## Conflict of Interest Statement

The authors declare that the research was conducted in the absence of any commercial or financial relationships that could be construed as a potential conflict of interest. The handling Editor declared a shared affiliation, though no other collaboration, with one of the authors CC.

## Footnotes

1 http://patternvision.com/

2 http://patternvision.com/

3 How to read the pattern tree graph: the left box of **Figures 5–8** shows the events occurring within the pattern, listed in the order in which they occur within the pattern. The first event in the pattern appears at the top and the last at the bottom. The lower right box shows the frequency of events within the pattern, each dot means that an event has been coded. The pattern diagram (the lines connecting the dots) shows the connection between events. The number of pattern diagrams illustrates how often the pattern occurs. Sub-patterns also occur when some of the events within the pattern occur without the whole of the pattern occurring. The upper box illustrates the real-time of the pattern. The lines show the connections between events, when they take place and how much time passes between each event.
